# Supplementary material for: Glucose Homeostasis, Metabolomics, and Pregnancy Outcomes After Bariatric Surgery (GLORIA): Protocol for a Multicentre Prospective Cohort Study
Source: J Clin Med. 2025 Jul 7;14(13):4782. doi: 10.3390/jcm14134782 (PMC12250646; doi:10.3390/jcm14134782)
Supplement: Supplementary file 1 [file jcm-14-04782-s001.zip › Table S1 - Clinical definitions and diagnostic criteria - kopie.pdf]

Table S1 Clinical definitions and diagnostic criteria considered in this study

Body mass index (BMI)	Weight (kg) / (height (m)) ²
Maternal age	Maternal age in early pregnancy (before 12 weeks)
Gestational age	Crown-to-rump length measured in the first trimester ultrasound performed before 12 weeks of gestation
Gestational weight gain	Difference between weight at first visit (before 12 weeks) and weight in last prenatal visit just prior to delivery
Gestational diabetes	Criteria used in the control group (1)<br>• Low risk women: 50 g GCT at 24-28 weeks of gestation using cut-off ≥ 130 mg/dl<br>• Women with prediabetes, obesity, or a history of GDM: 75 g OGTT at 24-28 weeks of gestation. Gestational diabetes is diagnosed if one or more values fasting or postprandial are above target: fasting blood glucose ≥ 92 mg/dl; 1h postprandial blood glucose ≥ 180 mg/dl; 2 h postprandial blood glucose ≥ 153 mg/dl (2013 WHO criteria criteria).<br><br>Criteria used in the bariatric surgery group (2)<br>Self-monitoring of capillary blood glucose for 3-7 days between 24 and 28 weeks of gestation. Gestational diabetes is diagnosed if 3 values fasting or postprandial are above target: fasting blood glucose ≥ 95 mg/dl; 1h postprandial blood glucose ≥ 140 mg/dl; 2h postprandial blood glucose ≥ 120 mg/dl (ADA criteria).<br>In case of suspicion of an unhealthy eating pattern involving excessive glucose intake, the treating physician may choose to optimize the patient's diet and then retest.
Gestational hypertension	≥ 20 weeks of gestation: blood pressure ≥ 140/90 mmHg *without* proteinuria or other signs/symptoms of preeclampsia-related end-organ dysfunction
Preeclampsia	≥ 20 weeks of gestation: new onset hypertension *and* proteinuria *and/or* significant end-organ dysfunction (acute kidney injury, liver dysfunction, neurological features, haemolysis/thrombocytopenia, fetal growth restriction).
Proteinuria	Proteinuria = dipstick ≥ 2+, ≥ 0.3 g protein/24 hours *or* ≥ 30 mg/dL protein in spot urine *or* spot urine protein/creatinine ratio ≥ 30 mg/mmol
Eclampsia	≥ 1 generalized convulsions and/or coma in the setting of preeclampsia and in the absence of other neurologic conditions
Haemolysis elevated liver enzymes and low platelets (HELPP) syndrome	Haemolysis with a microangiopathic blood smear, Elevated Liver enzymes, and a Low Platelet count (< 100.000 /µL)
Postpartum haemorrhage	Cumulative blood loss greater than 1000 mL with signs and symptoms of hypovolemia within 24 hours of the birth process, regardless of the route of delivery.
Miscarriage	Loss of pregnancy < 20 weeks of gestation
Stillbirth	Mors in utero > 20 weeks of gestation
Neonatal respiratory distress syndrome	Defined as at least 4 hours of respiratory support with oxygen, continuous positive airway pressure (CPAP) or intermittent positive pressure ventilation in the first 24 hours after birth.
Neonatal hypoglycaemia	Defined as symptomatic or asymptomatic glycaemia < 47 mg/dL
Neonatal hyperbilirubinemia	Severe hyperbilirubinemia defined as total serum bilirubin > 20 mg/dL
Polycythaemia	Defined as haematocrit of peripheral venous blood of > 65 %
Neonatal death	Defined as death within 28 days after birth
Neonatal intensive care unit (NICU) admission	NICU admission defined as requiring a duration of at least 24 h, duration of NICU admission
Preterm delivery	Deliver < 37 weeks
Macrosomia	Birth weight > 4 kg

Large for gestational age (LGA)	Gestational age adjusted birth weight > 90th percentile according to the standardized Flemish birth charts adjusted for parity and sex
Small for gestational age (SGA)	Gestational age adjusted birth weight < 10th percentile according to the standardized Flemish birth charts adjusted for parity and sex
Birth percentile	According to the standardized Flemish birth charts adjusted for parity, sex, and gestational age in weeks
Measured with bioelectrical impedance analysis (BIA)	Fat percentage (%), fat mass (kg), lean mass (kg), total body water (kg), dry lean mass (kg), total body water % (%), basal metabolic rate (kcal), BMI (kg/m^2)

1. Benhalima K, Minschart C, Van Crombrugge P, Calewaert P, Verhaeghe J, Vandamme S, et al. The 2019 Flemish consensus on screening for overt diabetes in early pregnancy and screening for gestational diabetes mellitus. Acta Clin Belg. 2019:1-8.
2. Benhalima K, Minschart C, Ceulemans D, Bogaerts A, Van Der Schueren B, Mathieu C, et al. Screening and Management of Gestational Diabetes Mellitus after Bariatric Surgery. Nutrients. 2018;10(10).
