# Supplementary material for: Glucose Homeostasis, Metabolomics, and Pregnancy Outcomes After Bariatric Surgery (GLORIA): Protocol for a Multicentre Prospective Cohort Study
Source: J Clin Med. 2025 Jul 7;14(13):4782. doi: 10.3390/jcm14134782 (PMC12250646; doi:10.3390/jcm14134782)
Supplement: Supplementary file 1 [file jcm-14-04782-s001.zip › Table S2 ENG_SELF-DESIGNED QUESTIONNAIRE ON USER-FRIENDLINESS OF THE MASKED CGM _v1_22-10-2021_non-bariatric group - kopie.pdf]

# SELF-DESIGNED QUESTIONNAIRE ON USER-FRIENDLINESS OF THE MASKED CGM

We are interested in your thoughts and feelings regarding your masked continuous glucose monitor (CGM). For each item below, please tick one box on each line that best indicates how much you agree or disagree with each statement as it pertains to your masked CGM. Don't leave any blank.

| **Using the masked CGM…** | **Strongly agree** | **Agree** | **Neutral** | **Disagree** | **Strongly disagree** |
|---|---|---|---|---|---|
| 1. Is uncomfortable or painful. | | | | | |
| 2. Makes it harder for me to sleep and/or disrupts my sleep. | | | | | |
| 3. Is discreet. | | | | | |
| 4. Causes more embarrassment about feeling different from others. | | | | | |
| 5. Makes me feel uncomfortable about how my body looks. | | | | | |
| 6. Causes other people to react negatively (e.g. to stare/ask intrusive questions). | | | | | |
| 7. Interferes a lot with daily life. | | | | | |
| 8. Interferes a lot with sports, etc. | | | | | |
| 9. Makes me feel like a robot/machine. | | | | | |
| 10. Makes me worry a lot. | | | | | |
| 11. Causes too many skin irritations (itch, swelling and/or redness) or bruises. | | | | | |
